# Antibacterial and anti-corona virus (229E) activity of *Nigella sativa* oil combined with photodynamic therapy based on methylene blue in wound infection: in vitro and in vivo study

**DOI:** 10.1186/s12866-023-03018-1

**Published:** 2023-09-29

**Authors:** Sahar E. Abo-Neima, Mostafa M. El-Sheekh, Mayasar I. Al-Zaban, Abeer I. M. EL-Sayed

**Affiliations:** 1 Physics Department, Faculty of Science, Damanhour University, Damanhour, El-Beheira Egypt; 2 Botany Department, Faculty of Science, Tanta University, Tanta, 31527 Egypt; 3 Department of Biology, College of Science, Princess Nourah bint Abdulrahman University, P.O.Box 84428, 11671 Riyadh, Saudi Arabia; 4 Botany and Microbiology Department, Faculty of Science, Damanhour University, Damanhour, El-Beheira Egypt

**Keywords:** Anti-coronavirus (229E), Antibacterial activity, Gas chromatography, Low-intensity laser therapy, *Nigella sativa*, Photodynamic therapy, *Staphylococcus aureus*, Transmission electron microscope

## Abstract

Microbial skin infections, antibiotic resistance, and poor wound healing are major problems, and new treatments are needed. Our study targeted solving this problem with *Nigella sativa* (NS) oil and photodynamic therapy based on methylene blue (MB-PDT). Antibacterial activity and minimum inhibitory concentration (MIC) were determined via agar well diffusion assay and broth microdilution, respectively. Transmission electron microscopy (TEM) proved deformations in *Staphylococcus aureus* ATCC 6538. Gas chromatography–mass spectrometry identified useful compounds that were suggested to be responsible for the potency of the oil. NS oil was tested as an antivirus against low pathogenic coronavirus (229E). Therapies examined, MB-PDT, NS, and MB-PDT + NS oil, to accelerate wound healing. The antibacterial efficacy against *S. aureus* was promising, with a MIC of 12.5% and TEM showing injured cells treated with NS oil. This oil inhibited 229E virus up to 42.85% and 32.14%. All tested therapies were successful in accelerating wound healing. The most successful was combined therapy (MB-PDT + NS oil), with a faster healing time. The combined therapy (MB-PDT + NS oil) reduced bacterial counts, which may be a key factor in accelerating wound healing. Skin wound histology was investigated; blood hematology and biochemical analysis did not change significantly after the safe combination treatment. A combination treatment could facilitate healing in a simple and inexpensive way in the future. Based on the results of the in vitro and in vivo studies, it was determined that NS oil had antibacterial and anti-corona virus activity when used in conjunction with photodynamic treatment based on methylene blue to treat wound infections.

## Introduction

Human ailments are reported to have been healed with numerous medicinal herbs throughout antiquity. Benhaddou et al. [1] reported that the role of these plants had increased significantly in recent years compared to other medicines owing to a few variables: low price, easy access without a prescription, the fact that natural remedies have fewer adverse effects, and the absence of a doctor's visit. Many investigations are needed to clarify the therapeutic characteristics, mechanism and mode of action, toxicity level, and safe use of diverse herbal medicinal plants to treat various ailments.

*Nigella sativa* (NS) seeds, referred to as black cumin, have shown a wide spectrum of pharmacological effects such as hepatoprotective [2], analgesic and anti-inflammatory, antipyretic [3], antitumor [4], antibacterial, antifungal [5], antiviral [6], immune stimulation [7]. Due to the distinctive features of *N. sativa*, it has been applied in treating many types of wounds and trauma [8]. NS is a potent source of nutritionally important components; its oil contains polyunsaturated fatty acids with other phytochemicals with strong antioxidant activity and hypoglycemic effects [9]. Hossain et al. [10] advocated for the use of NS due to its ability to lessen fatigue and boost energy levels. In 2020 alone, nearly 176 published research papers examined different areas of NS, of which about 17 articles related to the coronavirus disease (COVID-19).

The chemical constitution of essential oil (EO) is typically determined using gas chromatography-mass spectrometry (GC–MS). It is well recognized that various parameters, such as environmental circumstances, soil conditions, plant parts, herb species chemotypes, isolation techniques, etc., affect EO content and, consequently, their biological property. The chemical composition of NS seeds, which includes oils, proteins, carbohydrates, and fiber, was initially reported by Greenish et al. [11]. NS seed oil is used to preserve food as well as a spice, carminative, and painkiller. Because of the low toxicity of the seeds and their oil, NS seeds have also been reported to help prevent plaque and tooth decay. Over the past two decades [12], numerous studies have been conducted on the effects of black seed oil on a variety of physiological systems both in vivo and in vitro. Rathi et al. [13] observed that the seeds of the plant black cumin (BC) exhibited significant, encouraging antibacterial action, particularly against the gram-positive bacteria *Staphylococcus aureus* and *Listeria monocytogenes* and the gram-negative bacterium *Escherichia. coli*.

Coronaviruses (CoVs) with single-stranded positive-sense RNA genome and non-segmented nature are listed into four genera (alpha, beta, gamma, delta, and CoVs) according to phylogenetic clustering [14]. Antibiotics are often prescribed to treat bacterial respiratory infections. Moreover, they are given to treat potential secondary bacterial infections or to treat viral infections according to clinical antiviral indications. When antibiotics are misused to combat respiratory pandemic viruses, bacterial resistance emerges, and more people die. For this reason, we decided to test the antiviral effect of NS essential oil on the low pathogenic coronavirus 229E.

In this study, *N. sativa* oil was acquired from a local market in Alexandria, Egypt and used in a variety of experiments. Because of its low price, many people in our country use it on a daily basis; as a result, we have decided to purchase the essential oil from a local market in order to test its efficacy in a variety of contexts.

Injuries can occur in many other ways, including cuts from knives, falls, scrapes, and injuries from blunt instruments. Human wound healing is an incredibly complicated process that begins when an injury occurs by triggering many metabolic pathways in the wounded body [15]. Various bacteria often cause wound infections, which can cause severe inflammation and significantly slow the healing process. Serious microbial infections of wounds can sometimes cause permanent damage, potentially imposing a financial and psychological burden on patients and affecting their quality of life [16].

A wound is called physical damage that causes the skin to tear or open. Restoration of altered anatomical stability and compromised functional status of the skin depends on proper wound healing. A range of actions, including inflammation, cell proliferation, and cell type migration, lead to the repair of damaged tissue. Immediately after injury, the inflammatory stage begins with vasoconstriction that promotes homeostasis and produces inflammatory mediators [17].

Debridement, anti-inflammatory agents, and other treatments are typically used in clinical settings to treat skin wounds, improve wound healing, and prevent infection. In addition, antibiotics are often used to suppress bacterial inflammation and speed healing. However, misuse of antibiotics can have negative side effects and accelerate the emergence of antibiotic resistance [18]. In order to efficiently promote wound healing while being affordable, easy to use, and with few side effects, new technologies need to be developed.

In *Staphylococcus aureus*, superficial skin infections, antibiotic resistance, and poor wound healing are major problems, and new treatments are needed. A new therapeutic strategy for infections called antimicrobial photodynamic therapy (PDT) also accelerates wound healing in several different wound model organisms. PDT has been studied in tissue repair and in the treatment of uninfected or infected (due to their inactivation potential of microorganisms) skin wounds.

Public health is severely affected by wounds. Wound management failures leading to bacterial development and resistance can be caused by improper manipulation to promote wound healing and indiscriminate administration of antibiotics. Proper and appropriate approaches to wound care are critical. In addition, it is crucial to develop novel and efficient treatment approaches to reduce patient suffering, side effects, and infection-related mortality. Photodynamic treatment (PDT) could be a possible solution to this widespread health problem.

PDT is a versatile therapeutic approach that has been successfully used over the years to treat cancer [19, 20], age-related macular degeneration [21], and the inactivation of microorganisms in both clinical and non-clinical settings [22]. Recently, different research groups have been interested in investigating the strategy to improve wound healing/tissue regeneration after a traumatic lesion [23]. The conditions under which this therapeutic technique is used, in which a photosensitizer (PS) molecule/drug that can absorb visible light is brought into direct contact with the target tissue, affects the localized response to PDT treatment. Reactive oxygen species (ROS) are generated when PS is exposed to an appropriate wavelength of light in the presence of dioxygen (3O_2_). Different degrees of ROS can be generated depending on the PS structure and the light conditions [24]. Such levels of ROS may be toxic enough to promote i. destruction of malignant cells [25] or ii. Inactivation of pathogenic microorganisms, particularly multidrug-resistant strains [26], in situ, or at levels that cells can stimulate growth and tissue regeneration [27].

Methylene blue-based antimicrobial photodynamic treatment (MB-PDT) kills bacteria and promotes wound healing. We used a mouse superficial abrasion wound model to test the efficacy of MB-PDT and *N. sativa* oil in vivo by examining bacterial count, wound healing, and cosmetic outcomes. Mice with dorsal wounds and infected with *S. aureus* (strain ATCC 6538).

Experimentally, an infected wound was either left untreated (control) or treated topically with MB-PDT, *Nigella sativa* oil, or MB-PDT + *N. sativa* oil. According to this study, both therapies are noticeably successful: Better wound healing (reduction, disappearance of the crust), faster slight wound contraction after 24 h, and cosmetic results are all advantages of MB-PDT. The antibacterial activity of *N. sativa* oil is enhanced, although this does not appear to affect wound healing. The best clinical recovery has been shown in wounds treated with MB-PDT, but further research is needed to determine if more MB-PDT sessions, either alone or in conjunction with antibiotics, are beneficial in improving the microbicidal formulation of PDT. No synergistic antibacterial effect was found in this self-limiting infection model. Perhaps further research would be a good idea.

Acne vulgaris, rosacea, genital warts, and other inflammatory diseases have all been successfully treated with PDT [27]. ROS, which affect cell signaling and specifically cause cell damage or cell death in target tissues and microorganisms, are generated by photochemical reactive oxygen species (PDT) generation using a non-toxic dye known as a photosensitizer and specific wavelengths of safe light [28]. ROS plays a key role in regulating intracellular signaling. When determining the amount of ROS during photochemical reactions, the PDT dose is crucial [29]. Low-dose PDT affects proliferation and differentiation without significantly increased cell death [30], in contrast to the cellular toxicities caused by high levels of ROS, which promote differentiation of pluripotent stem cells such as mesenchymal stem cells, osteoblastic progenitor cells [29], neural stem cells [31] and others.

PDT is a form of clinical photochemotherapy in which a photosensitizer (PS) is introduced into target cells, followed by exposure to a specific wavelength light source. ROS and cell death are photo-induced when oxygen is present [30]. The treatment of local infections caused by bacteria, fungi, viruses, and parasites, and superficial malignancies has proven itself with this therapy [31]. PDT also effectively suppresses the microbial community within the biofilm [32].

Early wound healing significantly impacts the course and outcomes of future skin regeneration and the likelihood of pathologic scarring [33]. According to a recent study, the first phases of repair are when the benefits of PDT are most felt [34].

The current study's objectives were to assess in vitro antibacterial activity, MIC, along with taking some TEM micrographs illustrating the NS oil effects on bacterial pathogens and investigating the various components of this essential oil that are responsible for the destructive effect shown on the tested pathogens among exposure, via gas chromatography mass spectrometry analysis of *N. sativa* seeds oil. Then, study the impact of NS oil sample as an antiviral against low pathogenic coronavirus 229E. Also, study the antimicrobial effect of N. sativa essential oil with photodynamic therapy based on methylene blue in wound healing by *S. aureus* ATCC 6538 skin infection in vivo study. Finally, the main objective of the present work is to in vivo and in vivo study the antibacterial and anti-corona virus (229E) activity of *N. sativa* oil combined with photodynamic therapy based on methylene blue in wound infection.

## Materials and methods

Before and after each inquiry, the workstation bench was frequently sterilized and decontaminated with 70% ethanol. Glass objects in each experiment were wrapped in foil sheeting and autoclaved for fifteen minutes at 120 ^o^ C.

### Nigella sativa oil

*N. sativa* (NS) black seed oil, a product of El Captain Company (CAP PHARM) for extracting natural oils, plants, and cosmetics Egypt, was purchased from a local herbal medicine store in Alexandria, Egypt. The manufacturer claims it is a 100% pure natural product, cold-pressed oil without any additives.

### Assays on bacteria

#### Bacterial strains

This study used four referenced pathogenic bacterial strains: *S. aureus* ATCC 6538*, Listeria monocytogenes* ATCC 35152, *Salmonella typhimurium* ATCC 14028, and *Escherichia coli* ATCC 65922. The pathogenic bacterial culture was subcultured on Mueller- Hinton agar (MHA) and was set for incubation at 37 ^o^ C for 24 h. Subcultures were then kept at 5 ^o^ C, for further experiments.

#### Antibacterial assay of *N. sativa* essential oil

Using the agar well diffusion assay (AWDA), the antibacterial capacity of *N. sativa* oil against the above four clinically important bacterial pathogens was tested according to Er Kemal et al. [35]. At 37 °C, inoculate of the respective standard pathogenic bacteria with concentration between (1 × 10^8^) – (2 × 10^8^) (CFU/ml) were streaked onto (MHA) (DIFCO, USA) plates and by a sterilized cork porer a central well was made, 100 µL of oil sample was dropped into the well. Petri dishes were incubated at 37 °C for 18–24 h. The inhibition zone (IZ) around the well was measured to determine antibacterial activity. The inhibition was considered negative if there was no zone around the agar well. The antagonist potential was directly proportional to the IZ diameter [36]. The positive control was amoxicillin made from 500 mg tablets suspended in distilled water to a final stock concentration of 10 mg/mL and filter sterilized. The negative control was distilled water. The studies were carried out in triplicate, and the findings (mm) were reported as the mean and standard deviation of the inhibition zones (mm) [37].

#### Determination of minimum inhibitory concentration

To determine MIC of NS oil sample toward bacterial pathogens; the broth micro-dilution method was used. EO was first mixed with dimethyl sulfoxide DMSO (17 mg/mL, 99% purity) and then diluted in Muller Hinton broth (MHB). Briefly, 1.0 ml (MHB) was inoculated into all testing wells in 24 wells plates. 2.0 ml from the sample was dropped in the 1^st^ well (without being diluted), then to create a 1:2 dilution, 1.0 ml was aspirated and transferred to the following well, which had already been filled with 1.0 ml MHB, mixed well, then 1.0 ml was withdrawn from 1:2 dilution and dropped to the next 1.0 ml broth (1:4 dilution), this step was continued for preparing at least 8 dilutions for the remaining wells. 1.0 ml of bacterial inoculum (5.0 × 10^5^ CFU/well) was added to each well. A well served as a growth control well containing inoculated broth only was made in each sample/plate. A well served as negative control with only MHB was made in each sample plate. Plates were incubated at 35.0 ± 2.0 °C for 24.0 ± 2.0 h. After incubation, plates were kept in the dark to evaluate growth. All growth control wells have a turbid solution of growth, indicating test validity. All negative control wells were recorded as clear, indicating the accuracy of the test. After incubation, the appearance of turbid solution and a pellet on the bottom of the well was used to express bacterial growth. MIC was illustrated as the lowest concentration from EO without macroscopically seen growth [38].

#### Transmission electron microscopy

*S. aureus* ATCC 6538 cells were treated with an NS oil sample at Minimum Inhibitory Concentrations (MIC), this bacterial pathogen was identified due to its apparent and clear diameter of the zone of inhibition detected when treated with an NS oil sample among other pathogens tested, selected for a TEM. For the TEM preparation, from 24-h-old cultures grown on MHB medium, bacteria were extracted by centrifugation (at 4000 rpm for 10 min); the samples were then cleaned with distilled water, fixed in 3% glutaraldehyde, rinsed in phosphate buffer, and post-fixed in potassium permanganate solution for five minutes. at ambient temperature. The samples were dehydrated for 15 min in each ethanol dilution, ranging from 10 to 90%, and then for 30 min in absolute ethanol. Over a graded sequence, samples were infiltrated with epoxy resin and acetone until they were finally laid in plain resin. Using copper grids, extremely thin pieces were gathered. After that, sections were thrice stained with lead citrate and uranyl acetate. Stained sections were examined via JEOL-JEM 1010 transmission electron microscope at 70 kV at Alexandria University [39–41].

#### Gas chromatography analysis

Analysis of NS oil via GC was done in a Trace 1300 GC Ultra/Mass Spectrophotometer ISQ QD (Thermo Scientific) instrument, X-calibur 2.2 software (Thermo X-calibur), a device controlled by a computer at 70 electron volts. Using a micro syringe, one l μl of NS oil was introduced into the GC–MS, and scanning was conducted for 50–600 m/z at five scans per second. When separating the compounds, they were eluted from the column and then entered a detector which once a molecule was identified, could produce an electronic signal [42, 43]. From the injection, the time made (Initial time) till the elution happens is called the retention time (RT) [43, 44]. Before starting the GC–MS analysis, the temperature of the oven, gas flow rate used, and electron gun were programmed first. The oven temperature was adjusted at 50 ºC for 5 min then raised from 50 ºC to 200 ºC for 1 min (4 ºC/min), and 200–270 ºC (7 ºC/min) for 1 min. Helium gas (average velocity 39 cm/s) served as a carrier and an eluent. The helium gas flow rate was adjusted at 0.7 mL/min. 70 eV was the energy of the electrons liberated from the electron gun of the mass detector. The column employed here for separating components was A TG-5MS Zebron capillary column (length 30 m × 0.25 mm ID, 0.25 µm film thickness; Thermo) [45, 46].

#### Antiviral activity against low pathogenic coronavirus (229E)

Vero E6 cells were cultured for a day at a temperature of 37° C. in a six-well plate. The growth medium was eliminated from the cell culture plates, then the virus was plated (100 µL/well) and incubated for 1 h at 37 °C. Various concentrations of the examined compound (100 l/well) were inoculated for 1 h and incubated at 37 °C on the infected cells. After 1 h contact time, 1.5 ml of DMEM additionally with 2% agarose was loaded to the cell monolayer; Plates were allowed to set up and then kept warm at 37 °C until viral plaques formed (over 3 days). After two hours of formalin (10%) addition, plates were dyed with 0.1% crystal violet in distilled water [14, 47]. Plaques were then enumerated, and the percentage of plaque reduction versus the control was reported as in Eq. (1). Control wells contained untreated virus that was cultured with Vero cells.

1 $$\%\mathrm I\mathrm n\mathrm h\mathrm i\mathrm b\mathrm i\mathrm t\mathrm i\mathrm o\mathrm n=\frac{\mathrm{Viralcount}\left(\mathrm{untreated}\right)-\mathrm{Viralcount}(\mathrm{treated})}{\mathrm{Viralcount}(\mathrm{untreated})}\;x100$$

#### Animals and wound creation

All mice were of age 6–8 weeks; the mice were kept individually in cages for a few days prior to the experiment to prevent wound damage. The procedures were carried out in biosafety chambers. The study was done at the Faculty of Science at Damanhour University on mice that were aged from 6 to 8 weeks of both sex and weight between 35 ± 2gm. The mice were distributed in isolated metallic cages of size 15 × 10 × 8 under controlled conditions with standard food and water. Mice were intramuscularly anesthetized by infusion of sodium pentobarbital intraperitoneal. The dorsal region was cleaned with sterile 150 mM NaCl and 70% ethyl alcohol. A wound was made in mice skin by blunt-tipped scissors and dissection forceps. 0.1 ml from concentration 10^6^ CFU/ml of *S. aureus* of strain (ATCC 6538) suspension intraperitoneally except the control group received 250 µL of normal saline [48]. Twenty-four hours later specimen (blood) was taken from the animal’s tail and culture on a specific medium for *S. aureus* (Mannitol salt agar). Colonies growth was observed for *S. aureus,* indicating that animals are already infected with the test organism. Forty-eight hours later, each group received different treatments. Forty male albino mice that received wounds were distributed and divided into 4 groups (*n* = 10) for each group and were designed as follows:

- Group 1 (NCG): used as a negative control group of animals with no infected wounds and received 0.25 ml of normal saline daily.
- Group 2 (PCG): animals with infected wounds were used as positive control and cleaned using a saline solution.
- Group 3 (OG): animals with infected wounds and daily oral administration of 50 μL of black seed oil.
- Group 4 (MB-PDT G): animals with infected wounds were injected as group 3 then exposed the wounds to low-intensity laser therapy of power 2 mw for 10 min twice a week for two weeks.

To prevent external contamination, the treatment was carried out in a laminar flow over a period of 14 days. In addition, the weight and temperature of the mice were measured daily. Tissue samples were collected for histological examination, bacterial load assessment, and inflammatory mediator assessment. Blood samples were also collected for hematological markers.

#### Therapy protocols

After sensitization with MB, MB-PDT involved the light irradiation of diseased wounds (Sigma-Aldrich). As described in previous reports [49, 50], MB was soon made in the dark prior to application. A prepared MB stock solution was diluted to the required concentration with double distilled water. The infected wounds received two drops of MB and the wounds were then closed with adhesive plaster until the MB was fully absorbed. After incubation in the dark for 30 min, irradiation started [51]. Under inhalation anesthesia, each wound was illuminated with low-intensity laser radiation therapy and then black seed oil was applied topically to the wound daily. MB-PDT treatment protocol on *S. aureus* infected skin was compared to *Nigella sativa* oil-based treatments alone or in combination with *N. sativa* oil.

#### Low-intensity laser therapy and wound treatment

**Fig. 1:**
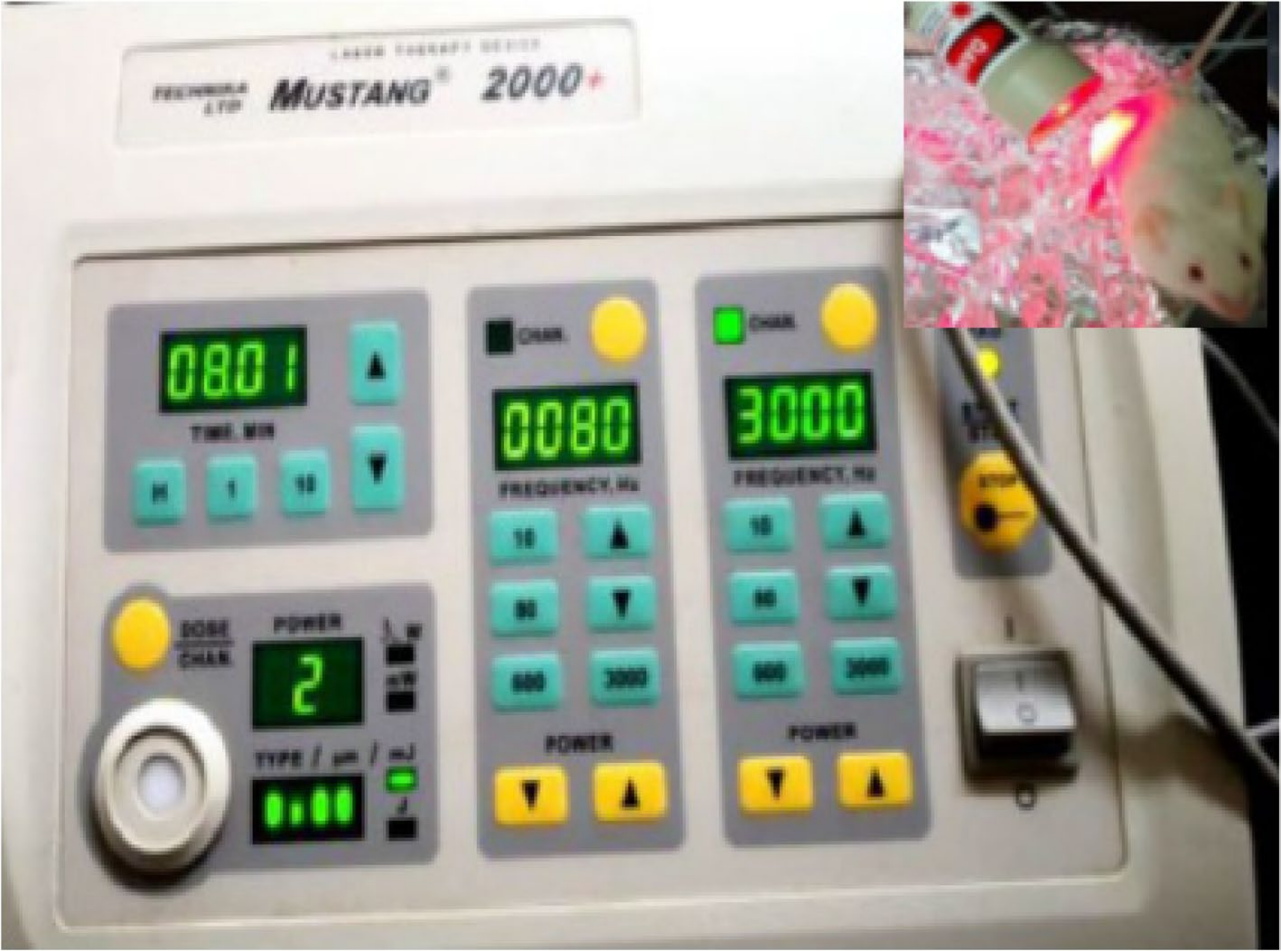
Photograph of low-intensity laser therapy device and laser probe irradiate the wounds of mice

A low-intensity laser treatment device (Mustang, 2000, Germany) with a maximum power of 2mw, emission frequencies in the range from 10 to 3000 Hz, and two outputs, which allowed the simultaneous connection of two laser transmitters, is shown in Fig. 1. Wounds were the laser probe was firmly placed and the wound was treated with the laser for 10 min.

#### Bacteriological examinations in tissues

The presence of the test strain in the blood is assessed qualitatively by streaking the samples directly onto selected media mannitol salt agar. After a 24-h incubation period at 37 °C, the plates were examined for the presence of *S. aureus.*

#### Hematology and clinical biochemistry of blood samples

Hematological measurements of blood were performed using an autoanalyzer, including hemoglobin (Hb), erythrocyte count (RBC), packed cell volume (PCV), and total leukocyte count (TLC). Both the differential leukocyte count (DLC) and erythrocyte sedimentation rate (ESR) were performed manually.

#### Histological analysis of wound skin

The mice were sacrificed after 14 days, and skin samples were taken from minor wounds at the affected sites. The removal of healthy skin tissue served as a control. Tissues were then dehydrated with gradient ethanol, rinsed in PBS, fixed at 4% polyformaldehyde, and embedded in paraffin wax. Hematoxylin and eosin were used to stain the paraffin slices after slicing them with a slicer (HE).

#### Analysis of biological safety of laser radiation

On day 14, mice in all groups received an injection of 1% pentobarbital sodium. Blood was drawn from the hearts of comatose mice using a syringe. The serum was then separated from the blood and used to test for biochemical indicators such as liver and kidney function, blood lipids, blood sugar, inorganic ions, and antioxidants. The blood was placed in a 1.5 mL centrifuge tube and kept at room temperature for two hours. The mice were then sacrificed, and the liver, spleen, and kidneys, which are the main internal organs, were removed and preserved in 4% paraformaldehyde, and serum samples were analyzed and histologically stained with H&E.

#### Excision wound model

Ketamine (30 mg/kg, ip) was used to anesthetize the mice before using a standard ring to mark a 500 mm^2^ area on the animal's back. The marked skin was then carefully sliced ​​across its entire thickness. On 1mm^2^ graph paper, wounds were recorded on the day of injury, every three days until day 20, and then every other day until healing was complete. Regular measurements of wound area were taken and wound contraction can be calculated from Eqs. (2) and (3).

2 $$\%\mathrm W\mathrm o\mathrm u\mathrm n\mathrm d\mathrm c\mathrm o\mathrm n\mathrm t\mathrm r\mathrm a\mathrm c\mathrm t\mathrm i\mathrm o\mathrm n=\frac{\mathrm{Healedarea}}{\mathrm{Totalwoundarea}}\;\mathrm x100$$

3 $$\mathrm{Healed area}=\mathrm{original wound area}-\mathrm{present wound area}$$

The significance in wound healing is determined by comparing the healed wound area of the test groups to the healed wound area of the control groups on the respective days. [17]

### Statistical analysis

The ANOVA test was used in the data processing. The mean standard deviation (SD) from at least three independent experiments is reported for each experimental result. Student's t-test was used to determine statistical significance. Statistics were considered significant when values between two independent groups were *p* < 0.05. [52]

## Results

### Antibacterial assay

**Fig. 2:**
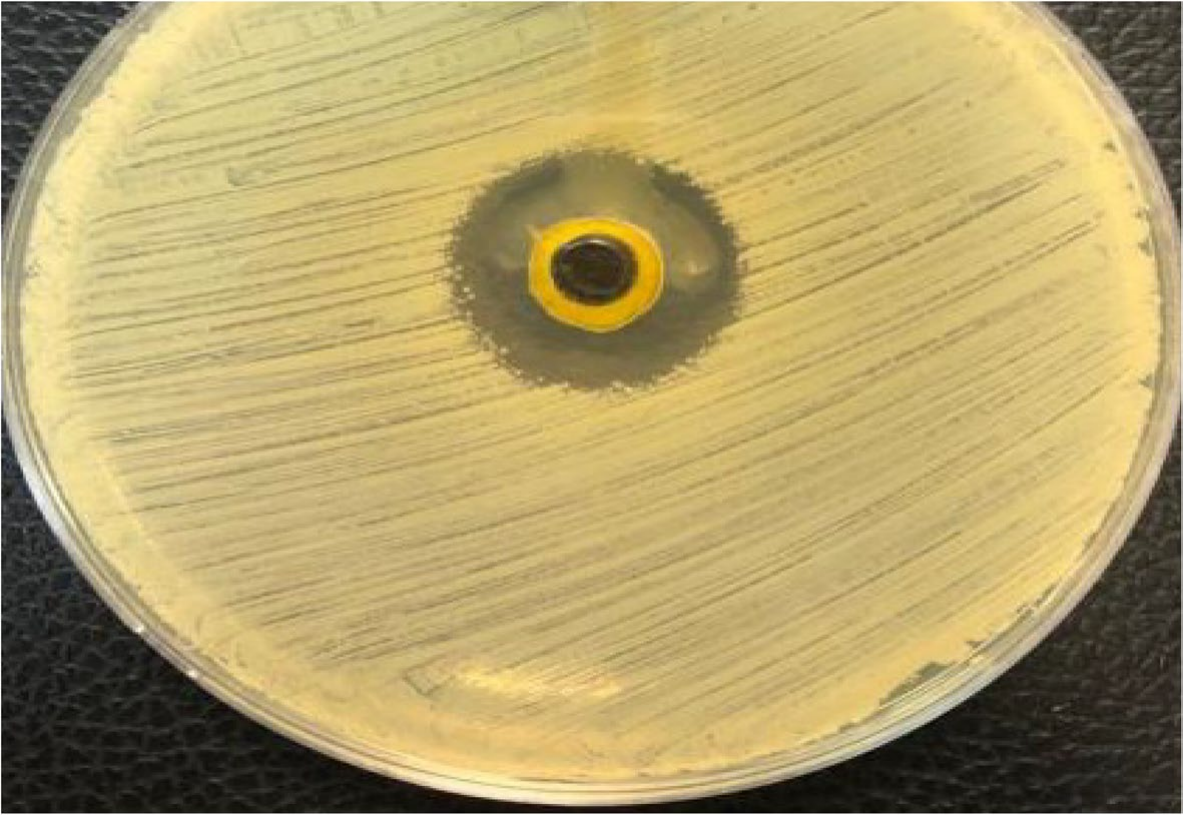
Antibacterial activity of *Nigella sativa* oil against *Staphylococcus aureus* ATCC 6538

Results obtained from *N. sativa* essential oil were done in triplicate and the average was taken to be collected in Table 1. The NS oil was very active and potent in inhibiting the growth of *S. aureus* ATCC 6538 as represented in Fig. 2 which illustrates the most obvious clear inhibition zone among all other pathogens tested, with mean inhibition diameter and standard deviations; 17.033 (SD = 0.80208) mm. this was followed by *E. coli* ATCC 65922 then *L. monocytogenes ATCC 35152*showing a clear zone of inhibition with calculated means and standard deviations, 14.033(SD = 0.30551) mm and 12.333 (SD = 0.25166) mm, respectively. But no inhibitory mode was detected against *S. typhimurium* ATCC 14028. The standard antibiotic used amoxicillin, 10 mg/ml, gave an inhibition zone larger than that of NS oil with all tested pathogens, as detected in Table 1.

**Table 1:** Antibacterial activity of *Nigella sativa* oil against bacterial pathogens

Bacterial strains	NS oil Mean of I.Z (mm) ± SD	Amoxicillin 10 mg/ml
*Staphylococcus aureus* ATCC 6538	17.033 $\pm$ 0.80208	20 ± 0.816497
*Escherichia coli* ATCC 65922	14.033 $\pm$ 0.30551	19.16667 ± 0.62361
*Listeria monocytogenes* ATCC 35152	12.333 $\pm$ 0.25166	18 ± 0.408248
*Salmonella typhimurium* ATCC 14028	NA	15 ± 0.408248

*NA* No Activity distinguished

### Determination of minimum inhibitory concentration

**Fig. 3:**
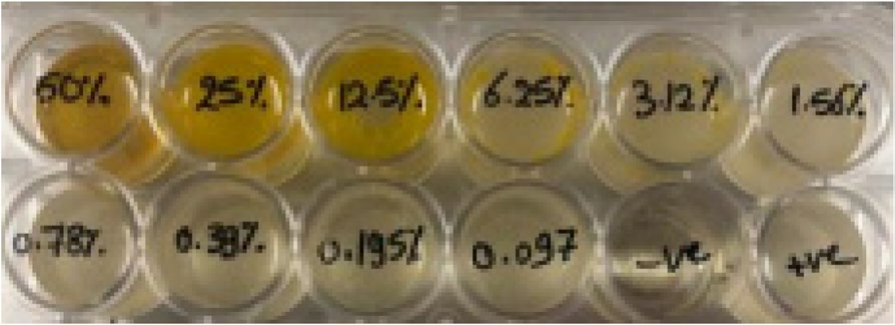
MIC reading results of *Nigella sativa* oil against *Staphylococcus aureus* ATCC 6538

MIC results of black seed oil toward all tested bacterial pathogens were summarized in Table 2. MIC exerted by the NS oil was seen to be 12.5, 25, and 50% against *S. aureus* ATCC 6538, which was shown in the reading results of (Fig. 3) *E. coli* ATCC 65922 and *L. monocytogenes* ATCC35152, respectively. The amount of oil needed to reach no visible growth towards tested pathogens was greater than those needed from the control antibiotic to achieve the same results.

**Table 2:** MIC of *Nigella sativa* oil against bacterial pathogens

pathogenic bacterial strain	MIC of NS oil (%)	MIC of Amoxicillin (%)
*Staphylococcus aureus* ATCC 6538	12.5	12.5
*Escherichia coli* ATCC 65922	25	6.25
*Listeria monocytogenes* ATCC 35152	50	6.25

### Transmission electron microscope

**Fig. 4:**
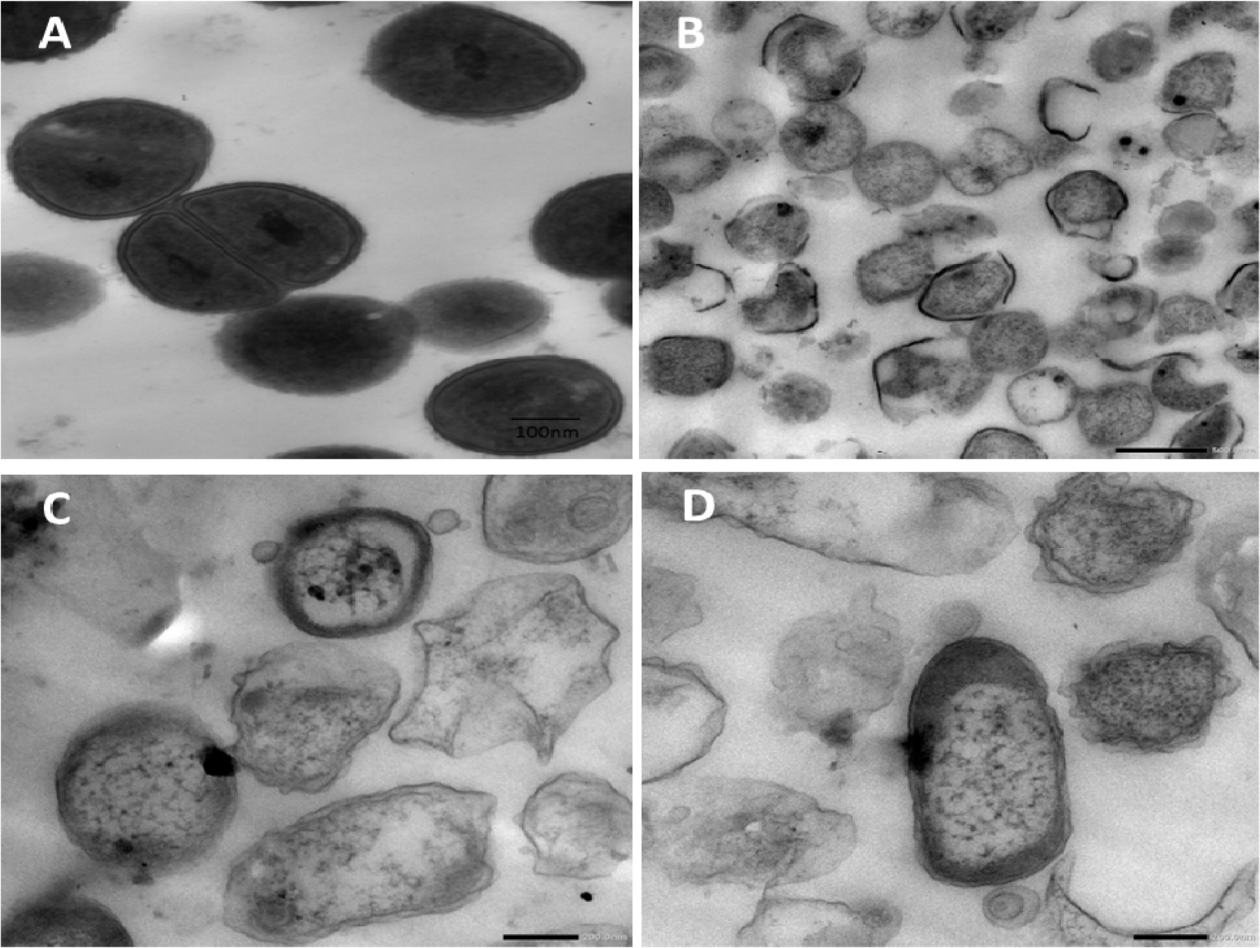
TEM images illustrated the changes in *Staphylococcus aureus* ATCC 6538 cells due to the strong inhibitory action of NS oil; **A** Control cell with normal appearance, **B**, **C**, and **D** treated cells with MIC of NS oil; cell walls were in a full destruction appearance leading to bacterial cell death. Due to the potent action of NS within the cell

The effect of Ns oil on the cells of *S. aureus* ATCC 6538 was shown in Fig. 4A**-**D. (Figure 4A) shows the *S. aureus* ATCC 6538 cells, before exposure to NS oil, seems to be in the division stage in a stable spherical to an oval shape, then from the monographs (Fig. 4B-D) cells were treated with NS oil sample at the (MIC), in which; severe dramatic changes were noticed with obvious cell destruction and damage in the whole cells were recorded.

### Gas chromatography analysis

**Fig. 5:**
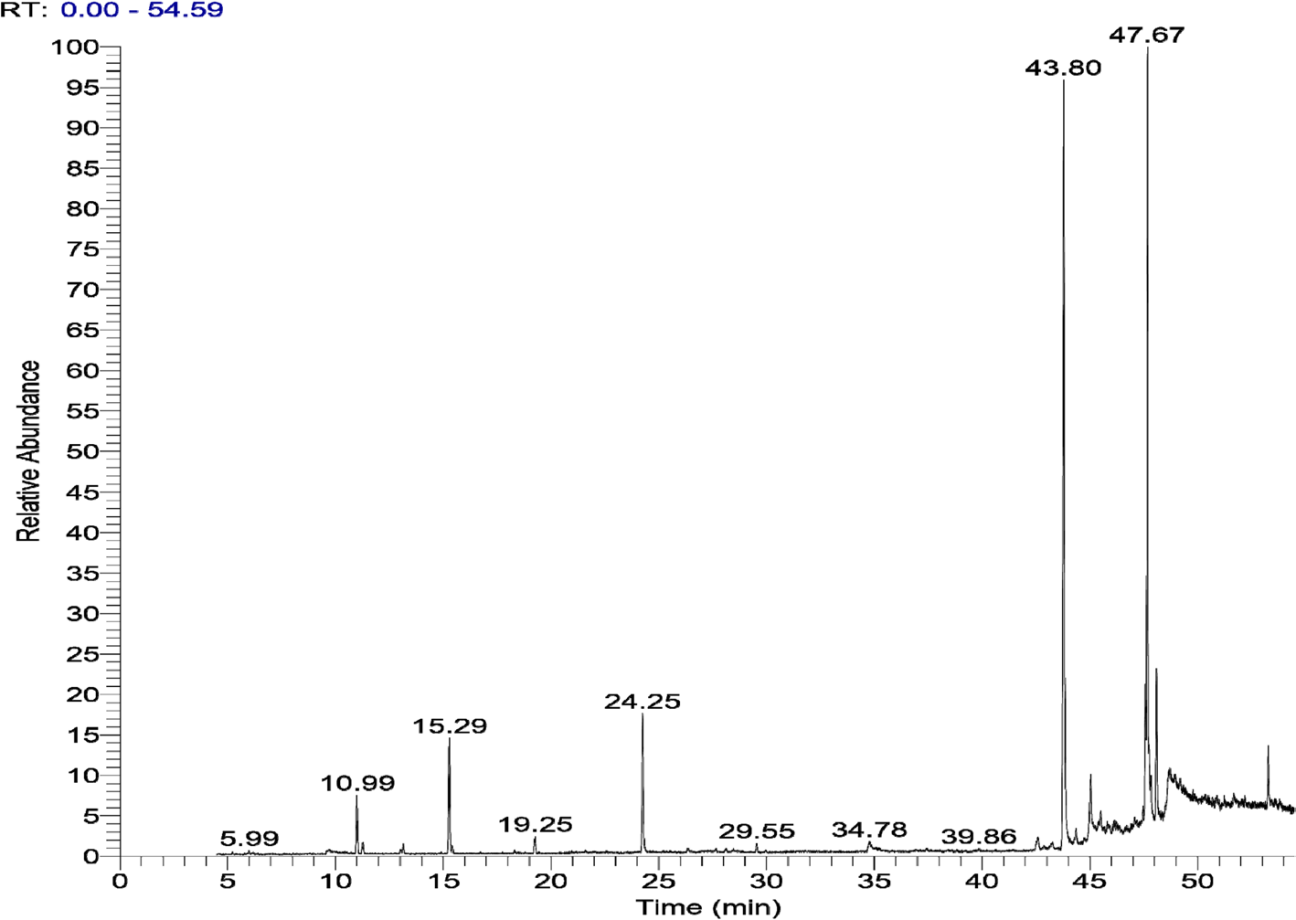
GC–MS chromatogram of *Nigella sativa* seed oil

This section outlined and illustrated the chemical constituents of the black seed oil sample. Names of these chemical compounds, their retention time, and area were listed in Table 3 and represented graphically in Fig. 5. The antibacterial activities of our sample against the tested pathogens might be attributed to the wide range of compounds included in our sample; which was also suggested in this study that the hard antimicrobial actions are due to; the presence of terpenes and others as shown in our GC analysis, led to membrane disruption shown in both Gram-positive and Gram-negative bacteria. The current inquiry may have experienced this type of disturbance. the current essential oil's diminished effectiveness against some bacteria may be due to the key ingredients' weak antimicrobial effects.

**Table 3:** Chemical composition of *N. sativa* essential oil analyzed by GC- MS

Phytochemical compounds	R.T. (min)	Area (%)
Nonadiyne	11.27	0.51
α-Pinene	13.15	0.44
p-Cymene	15.28	4.83
Camphene	15.43	0.21
Terpinen-4-ol	19.25	0.68
thujane	19.25	0.68
Thymoquinone	24.25	5.88
Longifolene	29.55	0.46
ß-Bisabolene	29.55	0.46
Mephenesin	34.79	0.60
Linolenic acid	34.79	0.60
Naphthalen-2-ol, 1-(5-thiazolyliminomethyl)-	43.26	0.44
carboxylic acid	43.26	0.44
Benzene dicarboxylic acid	44.35	0.72
Phthalic acid	45.03	2.71
Myristynoyl pantetheine	45.03	2.71
Erucic acid	45.50	0.85
Chlorpyrifos	45.82	0.58
Dasycarpidan	46.13	0.53
Eicosadienoic acid	46.24	0.28
Tri-pentaerythritol	46.63	0.20
14-Oxononadec-10-enoic acid, methyl ester	46.63	0.20
2-Myristynoyl pantetheine	46.72	0.26
Heptadecane,	47.45	0.33
Eicosenoic acid	47.83	0.41
Octadecenoic acid	47.83	0.41
Hexadecenoic acid	47.83	0.41
Methyl stearate	48.09	4.75
Oxiraneoctanoic acid	48.66	1.91
Oleic acid	48.66	1.91
Epoxypregnane	48.95	0.44
Piperidineacetic acid,	49.19	0.37
octanoic acid	49.34	0.19
Chromone, 5-hydroxy-6,7,8-trimethoxy-2,3-dimethyl	49.78	0.20
Nortazettine	49.86	0.17
Furandione	50.35	0.19
Piperidine acetic acid	50.69	0.25
Milbemycin B	50.91	0.63
Secoyohimban-19-oic acid	50.99	0.13
Deoxy spergualin	50.99	0.13
Enoic acid	51.23	0.31
Cyclohexane	51.67	0.60
17-Pentatriacontene	51.67	0.60
Oleic acid	52.20	0.28
Octadecane	52.20	0.28
Thio carbamic acid,	53.28	2.23
Silane, triethyl(2-phenylethoxy)	53.28	2.23
Gibberellic acid	53.59	0.36
Palmitic acid	53.81	0.31
[ n] phenanthrene-11-ol-2,3-dione-2a-propanoic acid lactone	53.81	0.31

### Antiviral activity of *N. sativa* oil against low pathogenic coronavirus (229E)

Our *N. sativa* oil sample was tested for its potency against low pathogenic coronavirus (229E). The result was detected at a concentration of 100 µl/ml and 10 µl/ml, the sample can in inhibit 229E virus up to 42.85% and 32.14%, respectively, as represented in Table 4.

**Table 4:** Antiviral activity of *Nigella sativa* oil against low pathogenic corona virus (229E)

Sample	Sample concentration (µl/ml)	Virus control (PFU/ml)	Virus titer and treatment (PFU/ml)	Viral inhibition %
*Nigella sativa* oil	100	3.26 × 10^5^	1.86 × 10^5^	42.85
	10		2.21 × 10^5^	32.14

### Skin wounds healing in mice

**Fig. 6:**
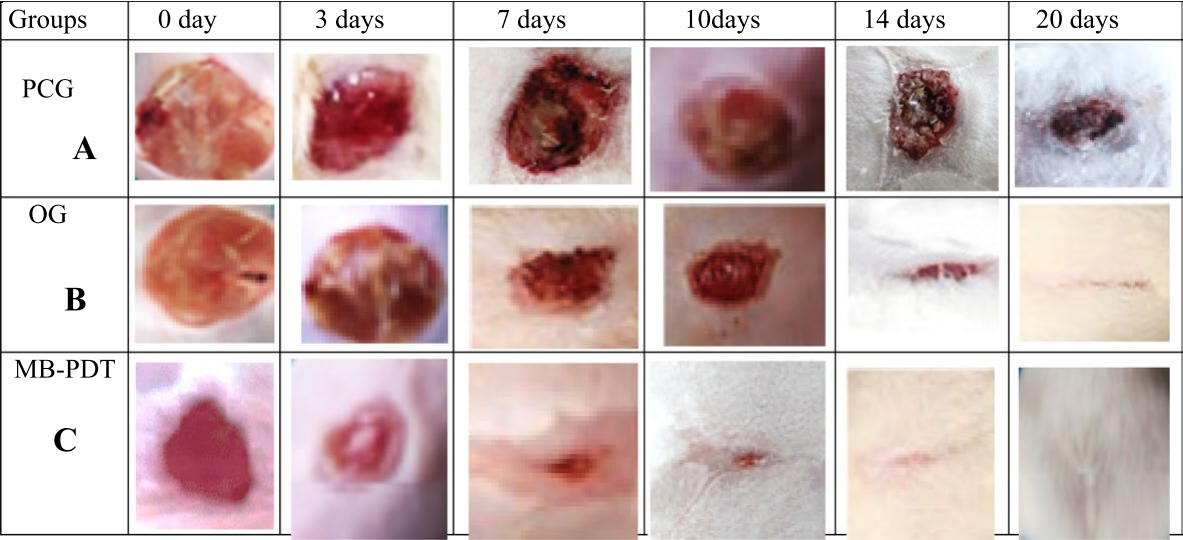
Development of the wounds through 20 days; **A** an infected rat without any treatment (PCG); **B** infected rat received *Nigella sativa* oil only (OG); **C** infected rat received *Nigella sativa* oil and exposed the wound to low intensity laser therapy

**Fig. 7:**
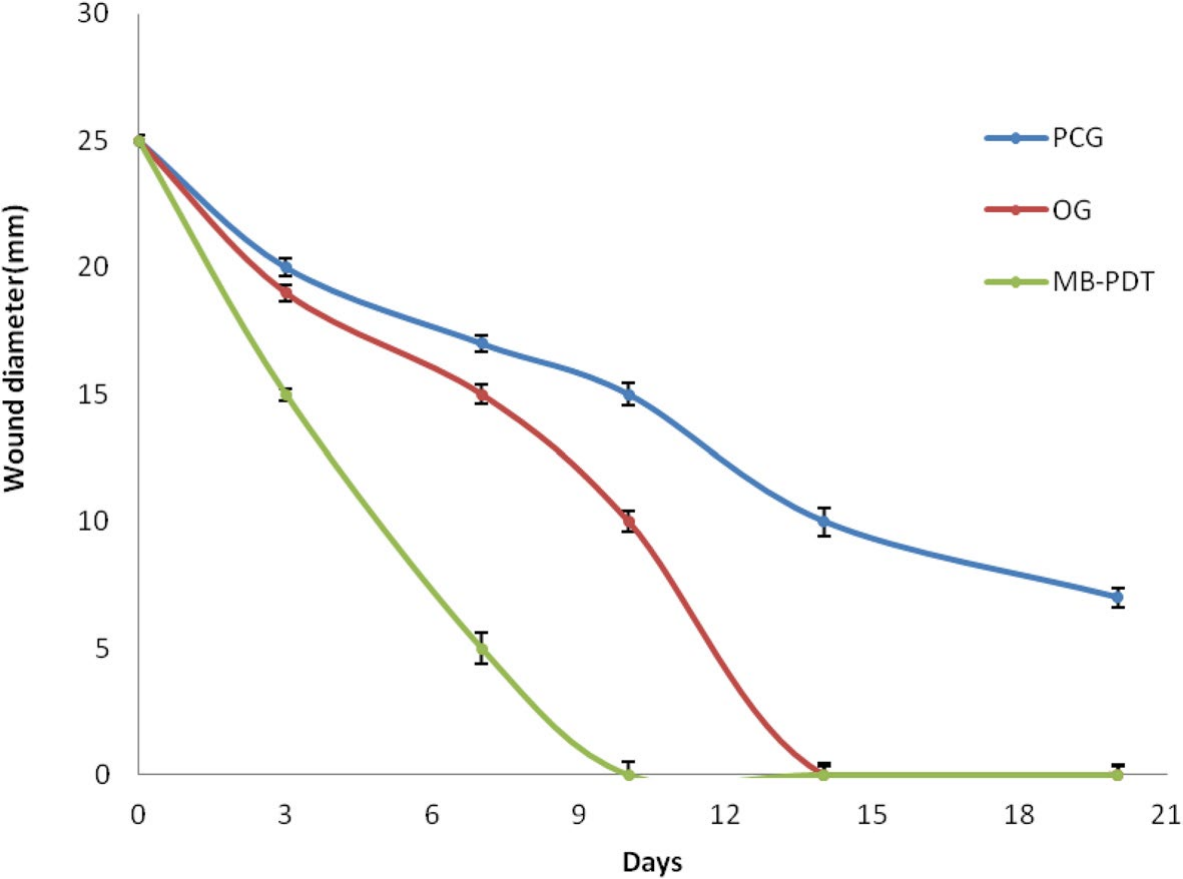
Diameter of the wounds through 20 days; (**A**) an infected rat without any treatment (PCG); (**B**) infected rat received *Nigella sativa* oil only (OG); (**C**) infected rat received *Nigella sativa* oil and exposed the wound to low intensity laser therapy

All mice groups were housed in the same breeding environment and given access to adequate food and water during the healing process. Mice received MB-PDT plus *N. sativa* oil in the treatment group and plain tap water in the control group, respectively. Figure 6 shows the results of wound healing. In mice from the treatment and control groups, the wounds were photographed at various time intervals, including days 0, 3, 7, 10, 14, and 20. (Fig. 6). Figure 7 shows the results of a statistical analysis of wound diameter at days 0, 3, 7, 10, 14, and 20 in mice from the treatment and control groups.

**Fig. 8:**
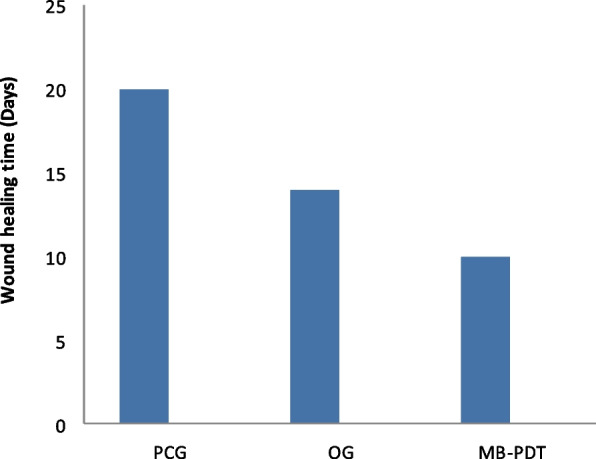
The wound healing time required for the wounds to completely heal for treated groups of mice as compared with control group

Figure 8 shows the results of a statistical analysis of wound diameter at days 0, 3, 7, 10, 14, and 20 in mice from the treatment and control groups as seen in Fig. 6. Over time, the wounds increased and the mice in the treated groups decreased in size and appeared smaller than the mice in the control group. On day 20, neither clearly visible wounds nor *N. sativa* oil + MB-PDT because wound healing occurs on day 10 with the combination treatment instead of day 14 with Nigella sativa oil alone, we concluded that combined treatment accelerated wound healing.

This suggests that combining treatments can promote wound healing in mice skin. As shown in Fig. 7 the wound diameters of the mice in the control and treated groups shrank over time, shrinking much more rapidly than in the control group. Figure 8 show that the time required for complete wound healing was much shorter in the treatment group compared to the control group. These results show how successful the combined therapy can be in accelerating the skin wound healing process in mice.

### Histology of skin wounds

Human wound healing is complicated [53]. Numerous elements, such as the wound, the bacteria, and the therapy settings, influence the healing process of wounds [54]. The MB-PDT effect in mice wound healing was investigated in this work using a wound infection model. Mice wounds treated with MB-PDT appear to reduce bacterial growth crucial for wound healing.

**Fig. 9:**
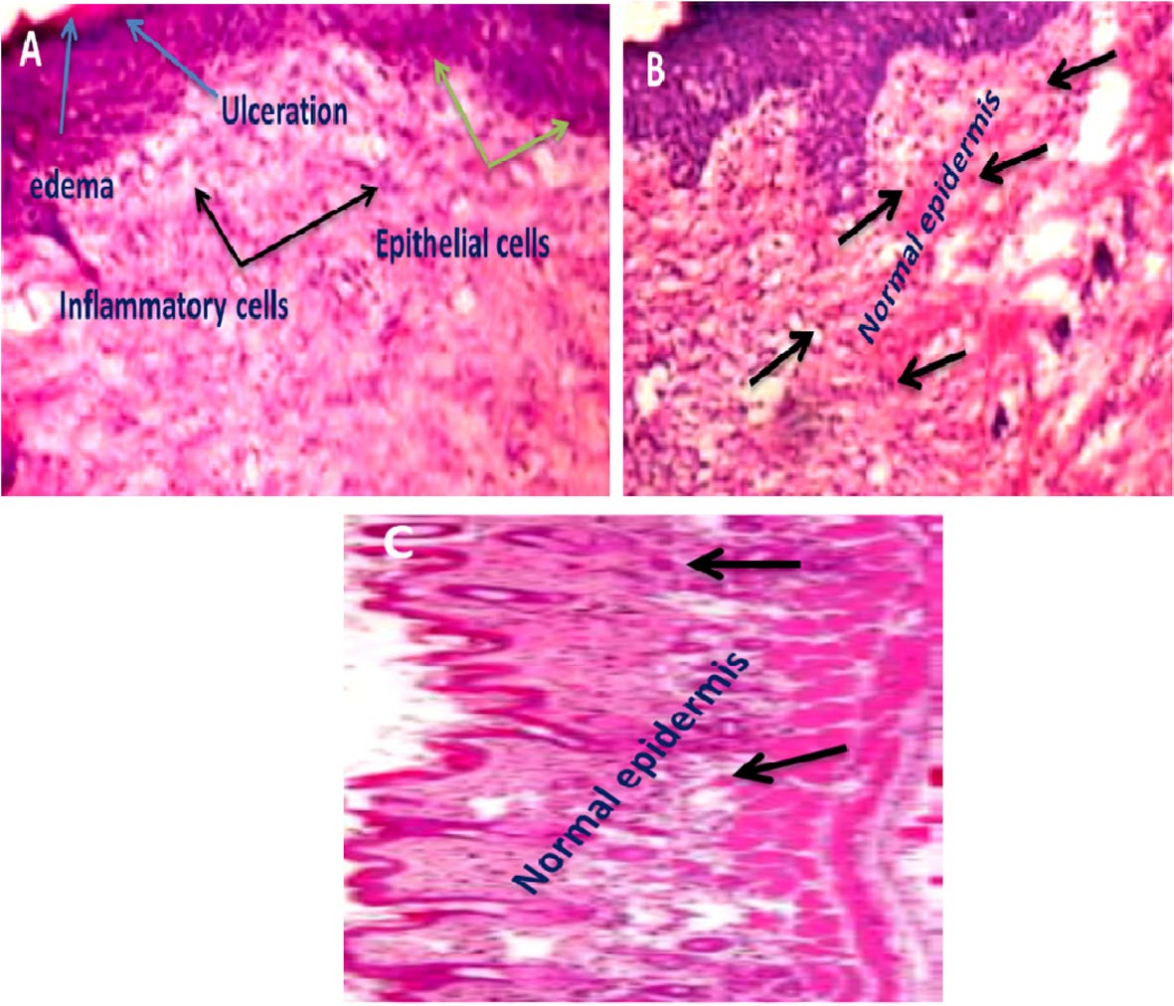
Histology of skin wounds in mice on day 20. The wounds skin tissue of mice from; (**A**) an infected mice without any treatment (PCG); (**B**) infected mice received *Nigella sativa* oil only (OG); (**C**) infected mice received *Nigella sativa* oil and MB-PDT were stained by hematoxylin and eosin H&E (400x)

Figure 9 shows the histology of the skin wounds for all groups. Blue and green arrows indicate ulcers and edema in the skin of control mice, while black arrows indicate early epithelialization and inflammatory cells (Fig. 9A). In the group treated with *N. sativa* oil, there were significantly fewer inflammatory cells in the skin-damaged tissue compared to the control group (Fig. 9B).

The skin wound tissue of the group treated with *N. sativa* oil, and MB-PDT shows healed skin structures with well-formed, almost normal appearing epidermis (Fig. 9C). This result agrees with the findings in (Figs. 6, 7 and 8).

### Blood hematology and biochemical analysis

The blood test known as the ESR, or Erythrocyte sedimentation Rate, is particularly effective at identifying infection or inflammation in the body. An abnormal ESR value can indicate whether there is inflammation in the body or not. High ESR levels are associated with conditions such as anemia and irregular weight loss/gain and can signal the appearance of malignant tissue. Low ESR values ​​result from sickle cell anemia, low plasma protein from liver or kidney disease, and other factors. Red blood cells (RBCs) eliminate extra carbon dioxide from the body while oxygenating it. A low RBCs count can indicate anemia or other diseases. Rarely circulatory disorders can result from an excess of red blood cells. The packed cell volume (PCV) refers to how many RBCs there are in a given volume of whole blood. A lower hematocrit can indicate excessive bleeding. You could also have an iron deficiency or another disease. Dehydration or other diseases may be responsible for elevated hematocrit. Red blood cells contain a protein called hemoglobin (Hb). It carries oxygen from the lungs to the other tissues of the body. Anemia to lung disease is just a few of the problems that abnormalities can indicate. Abnormalities can be a sign of problems ranging from anemia to lung disease. PCG exhibits significant changes in all tested hematological parameters, but the wound healing treatment by *Nigella Sativa* Oil alone or combined with MB-PDT exhibit no significant changes in hematological parameters. The treatment improves the state of blood circulation as shown in Table 5.

**Table 5:** Effect of different treatments on hematological parameters for wound healing mice

Blood parameters	NCG	PCG	OG	MB-PDT G
Hb (g/dl)	11.7 ± 0.22	6.3 ± 0.34	10.65 ± 0.27^**a,b**^	11.4 ± 0.34^a,c^
RBC / mm^3^	4.39 ± 0.52	8.13 ± 0.15	5.33 ± 0.14^**a,b**^	4.26 ± 0.37^a,c^
PCV (%)	35.88 ± 3.29	20.61 ± 1.23	33.42 ± 1.43^**a,b**^	34.12 ± 0.21^a,c^
TLC × 1000	5.43 ± 0.11	9.33 ± 0.24	6.78 ± 0.36^**a,b**^	5.56 ± 0.31^a,c^
ESR (mm/h)	3.66 ± 0.22	10.12 ± 0.47	4.71 ± 0.53^**a,b**^	3.22 ± 0.14^a,c^
Neutrophil (%)	11.75 ± 0.54	21.44 ± 0.32	15.11 ± 0.45^**a,b**^	10.39 ± 0.53^a,c^
Lymphocyte(%)	74.31 ± 0.31	87.96 ± 0.11	75.44 ± 0.33^**a,b**^	72.66 ± 0.55^a,c^
Monocytes (%)	6.13 ± 0.35	7.64 ± 0.27	6.88 ± 0.38^**a,b**^	5.76 ± 0.42^a,c^
Eosinophil (%)	3.75 ± 0.43	6.81 ± 0.13	5.66 ± 0.3icant 6^**a,b**^	4.11 ± 0.16^a,c^

Letters ^a^Not significant change as compared to NCG

^b^significant change as compared to PCG

^c^Very high significant change as compared to PCG

**Fig. 10:**
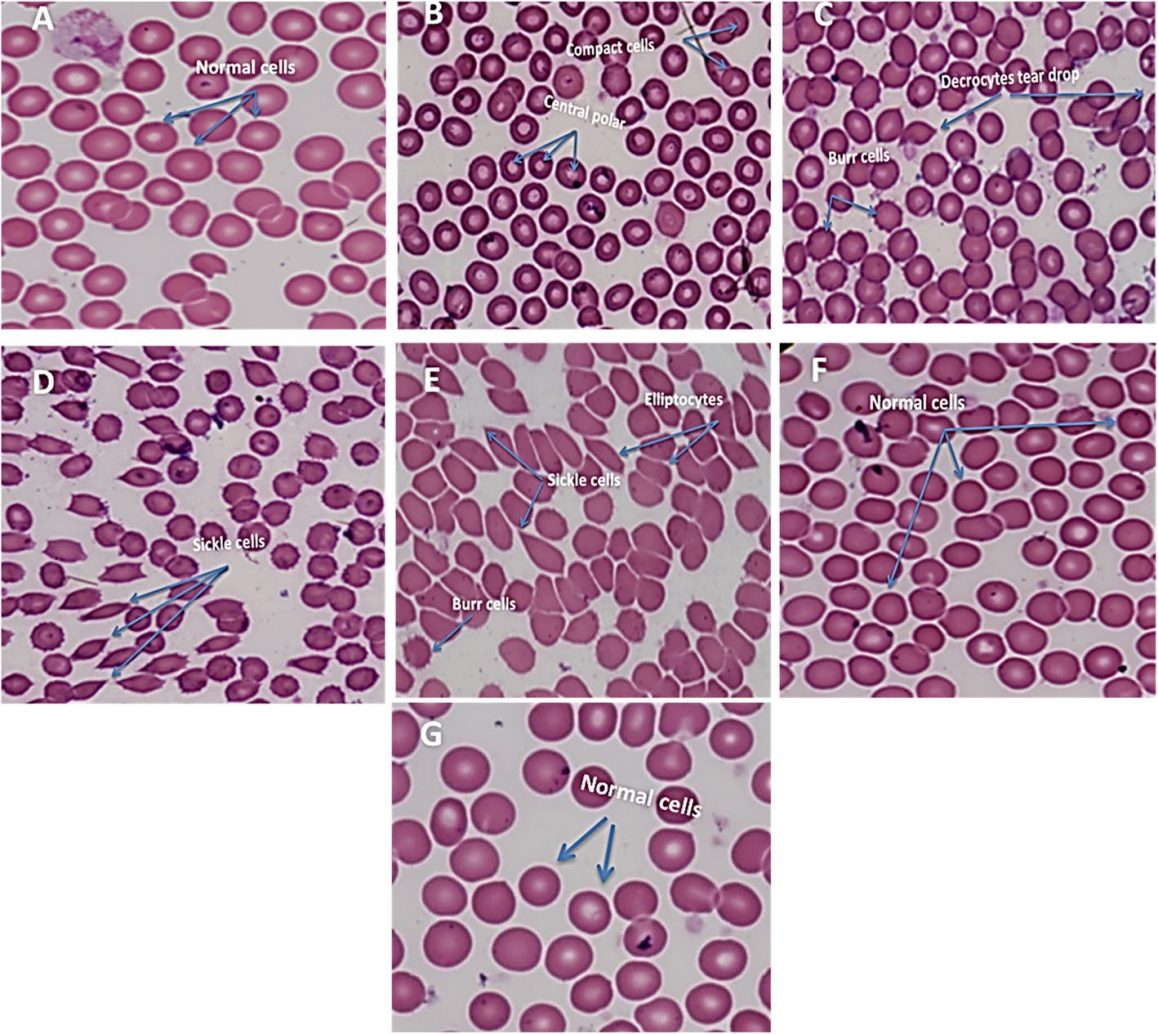
Blood smear test of mice from; (**A**) non infected mice (NCG); (**B**-**E**) infected mice without any treatment **(PCG**); (**F**) infected mice received *Nigella sativa* oil only (OG). **G**: infected mice received *Nigella sativa* oil and MB-PDT(400x)

We studied the shape of RBCs in Fig. 10. Blood smear tests of mice from non-infected mice (NCG), indicated normal RBCs with biconcave shape and that there is a mechanical stress and coloumb repulsive force that prevents cells from sticking together (Fig. 10A) infected mice without any treatment (PCG), which appears to be different types of anemic disease such as compact cells and central polar cells (Fig. 10B), Burr cells and Decrocytes teardrop (Fig. 10C), Sickle cell anemia, elliptocytes (Fig. 10D and E). According to the results in Table 6. Inorganic ions (potassium), blood lipids (total cholesterol), blood sugar (glucose), and antioxidant levels in mice were not significantly affected by MB-PDT (total superoxide dismutase).

**Table 6:** Biochemical analysis of blood

Test Parameters	PCG	OG	MB-PDT G
Total Superdioxide dismutase (U/mL)	329.66 ± 0.47	327.52 ± 0.76^NS^	326.98 ± 0.84^NS^
Potassium (mmole/L)	11.87 ± 0.12	10.56 ± 0.82^NS^	11.43 ± 0.53^NS^
Glucose (mmole/L)	7.55 ± 0.65	8.55 ± 0.31^NS^	9.78 ± 0.11^NS^
Urea nitrogen (mg/dl)	24.22 ± 0.16	23.95 ± 0.62^NS^	24.33 ± 0.74^NS^
Total cholesterol (mmole/L)	3.76 ± 0.66	4.99 ± 0.33^NS^	3.89 ± 0.45^NS^
Albmin (g/L)	25.14 ± 0.36	24.56 ± 0.22^NS^	25.65 ± 0.34^NS^
Aspartate aminotransferase (U/L)	242.26 ± 0.35	343.69 ± 0.47^NS^	345.91 ± 0.32^NS^

*NS* Not significant as compared to PCG

## Discussion

### Antibacterial assay

The most promising inhibition zone was observed to be 17.033 (SD = 0.80208) mm against *S. aureus* among all other examined pathogens. A good IZ was observed towards *E. coli* ATCC 65922 then *L. monocytogenes ATCC 35152,* while no inhibitory action was detected against *S. typhimurium* ATCC 14028. Similarly, our methods used to perform the different antibacterial experiments were used in the study done by Ramadan., [55], in which he tested the inhibitory effect of *N. sativa* seed extracts on *S. aureus* using the agar plate diffusion method. Investigations applying agar and broth dilution techniques have shown the antibacterial mode of NS extracts toward *S. aureus, S. epidermidis, E. coli*, and *P. aeruginosa*, as mentioned before [56, 57]. This antibacterial potential noticed in our investigations was also illustrated in the study done by Singh et al. [58] that most of the detected antimicrobial action in different EOs obtained from culinary along with spices herbs is strongly believed to originate from phenolic compounds, whereas other ingredients are known to barely share in antimicrobial activity. These were reinforced by the investigations of Arici, et al. [59], who stated that; the oil of *N. sativa* was active toward multi-drug resistant bacterial strains such as *S. aureus* and *Pseudomonas aeruginosa*, as well as having hard and promising antimicrobial activity mode against environmental pathogenic bacteria responsible for food spoilage. Additionally, Gawron, et al. [60] found that Poland-imported *N. sativa* oil was just as effective against methicillin-resistant *S. aureus* as it was against MRSA. Kemal et al. [35], 50 µl of 19% NS honey sample from Burdur region of Türkiye can inhibit S. aureus and *E. coli* by inhibition zone diameter of 23.00 ± 0.00 and 18.00 ± 1.40. Kooti et al. [61], supported our results by demonstrating the synergistic effects of NS extracts in eliminating *E. coli* and could minimize the growth of *L. monocytogenes* and *S. aureus*.

### Determination of MIC

The MIC was set to record: 12.5, 25, 50% against *S. aureus*, which was followed by *Escherichia coli* and *Listeria monocytogenes*, respectively. In accordance, Gawron et al. [60] found that; the MIC of *N. sativa* oil brought from Poland was tested against *S. aureus,* and the results ranged from 40 to 160 μg/ml.

### Transmission electron microscopy

Our images taken of *S. aureus* when treated with NS oil demonstrated how NS causes cell death by rupturing the *S. aureus* cell wall; these effects were amplified and can be referred to as bactericidal activity. This opinion was strongly supported by the findings mentioned by Uzair, et al. [62]; the ultrastructural changes in the MRSA isolates when using NS, demonstrated cell death and cell surface damage in the *S. aureus* cells.

### Gas chromatography analysis

Also, our NS oil sample detected the presence of longifolene, which is effective as an insect repellent. GC–MS analysis in this study listed a mixture of saturated fatty acids as; Palmitic acid, stearic acid, myristic acid, stearic acid, oleic acid, arachidonic acid, linoleic acid, eicosadienoic acid, which play an important role as immune boosting, anti-inflammatory and maintain skin moisture. The major terpenes as; α-pinene, thymoquinone (TQ), p-cymene, have been identified in our NS sample. According to this study, the current essential oil's diminished effectiveness against some bacteria may be due to the key ingredients' weak antimicrobial effects. In our NS oil via GC analysis, Oleic acid and linoleic acid are found; that can modulate cardiac glycosides' interaction with the sodium pump, as stated by Hossain, et al. [10]. That is in accordance with what was mentioned in the study done by Ahmad, et al. [63]; thymohydroquinone has a highly remarkable action toward many multi-drug-resistant, including *S. aureus* as an example of gram-positive microorganisms and gram-negative bacteria such as *E. coli*. Thymoquinone, *N. sativa*'s primary quinone component, is what gives the plant its medical value, for its different attractive pharmacological properties including hepatoprotective, anti-inflammatory, antibacterial, antioxidant, fungicidal, nephroprotective, anticancer (raising the activities of glutathione transferase and quinine reductase and acts as a strong potential prophylactic source toward toxicity in hepatic cancer and chemical carcinogenesis), along with improving hyperlipidemia and defend against the development of atherosclerosis also, this ingredient have valuable results for hyperglycemia and hyperlipidemia. These were also clear in the GC–MS study done by Singh et al. [58]; that the essential oil contained remarkable amounts of phenolics (thymol and carvacrol) and thus, the antimicrobial action might be for these compounds with p-cymene. Also, the potency of inhibition and the antimicrobial spectrum of the NS oil suggested that many complex reactions among individual components resulted in the overall activity. Proteins can be alkylated by aromadendrene, which changes the proteins' structure. Moreover, the chemical modification (such as oxidation) of EO components may be a factor in how effective they are against bacteria. Citral, d-limonene, and α-pinene have already been oxidized in the air, and their "products" have helped to increase the antibacterial action as reported by Horváth, et al. [64].

### Antiviral activity of *N. sativa* oil against low pathogenic coronavirus (229E)

Ns oil sample in our study had a good potency in inhibiting the low pathogenic coronavirus (229E), Similarly, Horváth et al. [64] reported in their review that; essential oils appear to have a lot of possible cellular targets due to their large number of constituents, and they may be utilized to treat a variety of disorders of the upper and lower respiratory tract. Transient receptor potential (TRP) ion channels in the airways are another site of interaction with EO components. According to sequence homology, the TRP superfamily of cation channels is classified into six subfamilies: TRPC (canonical), TRPV (vanilloid), TRPM (melastatin), TRPA (ankyrin), TRPP (polycystin), and TRPML (mucolipin). These ion channels are believed to be crucial in developing respiratory conditions such as asthma, chronic obstructive pulmonary disease (COPD), and cough.

In accordance with our results, Koshak et al. [65], mentioned that a number of viruses including Coronavirus infection is being treated and their replication is inhibited, with complementary herbal medicines, which contain a variety of biologically active components as in *N. sativa.* These treatments are frequently used to treat respiratory conditions and flu symptoms. Due to its numerous pharmacological actions, including its anti-inflammatory, anti-viral, and immunomodulatory effects, *Nigella sativa L.* is specifically recommended as a possible phytomedicine. An in vitro experiment revealed that *N. sativa L*. reduced the coronavirus burden.

### Skin wounds healing in mice

MB-PDT was better than *N. sativa* oil treatment in terms of wound healing rate and cosmetic outcome. This study was done by Topaloglu et al. [66] and crust loss in days shows clear differences in favor of PDT, as reported by Dai et al. [67]. Not only changes in wound size but also significant contraction on the first day after MB-PDT compared to untreated wounds.

### Histology of skin wounds

Histopathology is the gold standard method for measuring wound healing and determining epidermal re-epithelialization [68]. We observed several histological changes as wound healing progressed, such as thinning of the epidermis, elongation of the epithelial core, swelling of the dermis with collagen changes, and infiltration of inflammatory cells, as reported by Dai et al. [67]. The combined therapy significantly reduces the bacterial load on the wound, which minimizes tissue inflammation and accelerates the healing process. Due to the antioxidant, it contains, *Nigella Sativa* Oil supports wound healing by reducing inflammation and the presence of bacteria, which promotes the growth of body tissues and accelerates the healing process. PDT was performed in multiple steps and accelerated tissue repair, leading to good results in wound healing. PDT has shown potential as a therapeutic approach, preventing bacterial regrowth, or killing bacteria and significantly accelerating wound healing.

PDT acted in multiple steps and accelerated tissue repair, leading to good wound healing results. PDT has shown potential as a therapeutic approach because it can kill or stop bacteria from growing, which significantly accelerates wound healing.

### Blood hematology and biochemical analysis

Infected mice that received *Nigella sativa* oil only (OG) and infected mice that received *Nigella sativa* oil + MB-PDT appeared an improve in the shape of RBCs and seemed to be normal. This is an indicator that the combined therapy repairs the blood cells to their normal shape. Since the diameter of rat erythrocytes is in the range of 12–16 µm and the blood capillaries are in the range of 6-8 µm, the erythrocytes must be folded to pass through the blood capillaries to carry out metabolic processes [69]. The sticking together of neighboring cells in such a way that they share a common membrane and/or the loss of cell membrane elasticity, which impairs the folding mechanisms, will not allow the erythrocytes to pass through the blood capillaries and thus not carry out metabolic processes. Measuring the electrophoretic mobility of erythrocytes is now the most convenient method for estimating these charges both in experimental studies and in clinical practice [70]. Also, as results of combined therapy indicated improvement in the biochemical analysis of blood seems to be normal as compared with the untreated wound healing group.

## Conclusions

Our experimental studies indicated that many pathogens strongly resist *N. sativa* oil. *N. sativa* essential oil was considered powerful for its important antibacterial activity, MIC. The study also showed, using TEM images, that NS induced bacterial cell wall disruption, which may have had a bactericidal effect, as demonstrated by lysis and disruption of the *S. aureus* cell wall. All these findings hardly support the traditional applications of *N. sativa* in various fields. Also, the GC analysis and the anti-corona (299E) virus results performed in our study reinforced the influence of this herb pharmacological active ingredients to face life-threatening diseases, including the coronavirus. For that, the daily usage of *N. sativa* in adequate amounts does not require any physician prescription or approval from any health governing agency. In the present study, it was concluded that from in vivo study in contrast to self-healing, the three therapy methods examined, MB-PDT, *Nigella Sativa* Oil, and MB-PDT + *N. sativa* Oil, were successful. The greatest reduction of *S. aureus* was observed with *N. sativa* Oil; however, MB-PDT outperformed it regarding wound healing speed and aesthetic results. In terms of wound healing time and aesthetic results, MB-PDT outperformed *Nigella Sativa* Oil treatment. This reduction in wound size and scab removal over time clearly favors PDT. In addition to size changes, there was a noticeable contraction on the first day after MB-PDT compared to untreated wounds.

*Nigella sativa* oil can significantly help accelerate wound healing depending on metabolic conditions. Its anti-inflammatory, tissue growth stimulating, and antioxidant properties may also have a therapeutic effect on skin wound healing. When comparing the two treatments, MB-PDT + *Nigella Sativa* Oil outperformed MB-PDT or *N. sativa* oil alone. With the combined therapy (MB-PDT + black cumin oil), the skin lesions of the mice healed faster than the control. The combined therapy reduced bacterial counts, which may be a key factor in accelerating wound healing. Blood hematology and biochemical analysis did not change significantly after the safe combination treatment. The combination treatment could facilitate healing in a simple and inexpensive way. It was concluded that *N. sativa* oil combined with photodynamic therapy based on methylene blue in wound infection: In vitro and in vivo study has antibacterial and anti-corona virus activity.

## Data Availability

The datasets used and/or analyzed during the current study are available from the corresponding author on reasonable request.
